# Trauma and Religious Complexity in Later Life

**DOI:** 10.1093/geroni/igaa057.1339

**Published:** 2020-12-16

**Authors:** Rennae Wigton, Shannon Jones, Austin Prusak, Andrew Futterman

**Affiliations:** 1 Loyola University Maryland, Butler, Pennsylvania, United States; 2 Loyola University Maryland, Baltimore, Maryland, United States

## Abstract

The present study examines the impact of traumatic life events on religious complexity in later life. We anticipated that those older adults experiencing stressors that produce significant personal vulnerability (e.g., life threatening illnesses) demonstrate reduced complexity of belief and behavior (e.g., less belief with doubt). From a sample of 278 semi-structured interviews of older adults (aged 55-101 years-old.) from six New England and New York states, we analyzed 166 interviews using grounded theory (Strauss & Corbin, 1990). Individuals who experienced trauma related to war, close familial loss, and/or severe physical illness tended to be “true believers,” (i.e., adhere to rigid belief orthodoxy; Hoffer, 1950). By contrast, those who experienced less severe trauma (e.g., minor illness, job loss) were less apt to describe rigid belief. Temporal proximity of trauma was not consistently associated with greater complexity of belief and behavior, in the sense that with great distance from trauma, individuals were able to “work through” their experiences of trauma, and thereby increase complexity of belief and behavior. This is consistent with findings by Harris and Leak (2015), Krause and Hayward (2012), and Wong (2013) that suggest that trauma leading to personal vulnerability leads to long-term physical, mental, behavioral, and spiritual deficits that rigid religious belief and behavior help to offset. These findings are discussed in terms of psychological theories of grief resolution, personal coping, and terror management.

